# DNA-controlled protein fluorescence: Design of aptamer-split peptide hetero-modulator for GFP to respond to intracellular ATP levels

**DOI:** 10.1093/nar/gkae532

**Published:** 2024-06-25

**Authors:** Ki Sung Park, Hanvit Cha, Jia Niu, Hyongsok Tom Soh, Jin Hyup Lee, Seung Pil Pack

**Affiliations:** Department of Biotechnology and Bioinformatics, Korea University, Sejong 30019, Republic of Korea; Biological Clock-based Anti-Aging Convergence RLRC, Korea University, Sejong 30019, Republic of Korea; Biological Clock-based Anti-Aging Convergence RLRC, Korea University, Sejong 30019, Republic of Korea; Department of Food and Biotechnology, Korea University, Sejong 30019, Republic of Korea; Department of Chemistry, Boston College, Chestnut Hill, MA 02467, USA; Department of Electrical Engineering, Stanford University, Stanford, CA 94305, USA; Department of Radiology, Stanford University, Stanford, CA 94305, USA; Biological Clock-based Anti-Aging Convergence RLRC, Korea University, Sejong 30019, Republic of Korea; Department of Food and Biotechnology, Korea University, Sejong 30019, Republic of Korea; Department of Biotechnology and Bioinformatics, Korea University, Sejong 30019, Republic of Korea; Biological Clock-based Anti-Aging Convergence RLRC, Korea University, Sejong 30019, Republic of Korea

## Abstract

Enabling the precise control of protein functions with artificially programmed reaction patterns is beneficial for investigating biological processes. Although several strategies have been established that employ the programmability of nucleic acid, they have been limited to DNA hybridization without external stimuli or target binding. Here, we report an approach for the DNA-mediated control of the tripartite split-GFP assembly via aptamers with responsiveness to intracellular small molecules as stimuli. We designed a novel structure-switching aptamer-peptide conjugate as a hetero modulator for split GFP in response to ATP. By conjugating two peptides (S10/11) derived from the tripartite split-GFP to ATP aptamer, we achieved GFP reassembly using only ATP as a trigger molecule. The response to ATP at ≥4 mM concentrations indicated that it can be applied to respond to intracellular ATP in live cells. Furthermore, our hetero-modulator exhibited high and long-term stability, with a half-life of approximately four days in a serum stability assay, demonstrating resistance to nuclease degradation. We validated that our aptamer-modulator split GFP was successfully reconstituted in the cell in response to intracellular ATP levels. Our aptamer-modulated split GFP platform can be utilized to monitor a wide range of intracellular metabolites by replacing the aptamer sequence.

## Graphical Abstract

**Graphical Abstract:**
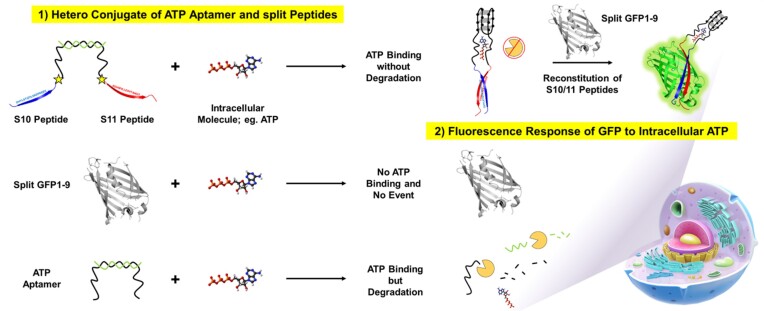

## Introduction

In crowded cellular networks, proteins play pivotal roles that sophisticatedly carry out essential functions involved in a wide range of biological activities, including catalyzing chemical reactions (*e.g.*, enzymes), transporting molecules (*e.g.*, hemoglobin), providing structural support (*e.g.*, collagen), and facilitating cell signaling (*e.g.*, receptors) (1). The structure of a protein is directly related to its function. For example, the specific shape of the active site is formed for substrate binding or chemical reactions, and conformational changes occur in response to environmental cues or binding to other molecules to open or close channels. These different protein structures and functions are completed by dynamic interactions with intracellular DNA, RNA, proteins, and low-molecular-weight ligands to form interwoven molecular reaction networks triggered by environmental, physical, or chemical stimuli (2–4).

Inspired by nature, extensive research efforts have been devoted to developing artificially programmed reaction patterns within dynamic interactions. The precise control of their function provides significant benefits in investigating biological processes such as signal transduction, gene suppression, and genome editing (5). Attractive strategies have demonstrated that the utilization of DNA as a template to control protein function allows for sophisticated tunability by sequence-specific hybridization, taking advantage of the high programmability of nucleic acids. One example of DNA-templated control of protein activity is a DNA-guided multiple enzyme cascade reaction. This strategy involves fabricating artificial multi-enzyme complexes (MECs) using complementary single-stranded DNA as a template to attach two different enzymes. Hybridization between two DNA strands brings these attached enzymes into proximity, accelerating the multistep catalytic reaction (6,7). In addition, DNA-templated allosteric regulators have been employed to control inactive enzyme-inhibitor complexes using programmed DNA sequences, turning on the enzyme activity by releasing the inhibitor by adding an activator strand (8).

Although these strategies have been proven advantageous for DNA programmability, the control of protein function has been limited to double-stranded DNA hybridization without external stimuli to direct the proximity between binding partners. An alternative approach is to use DNA aptamers as warheads to respond to environmental stimuli. Aptamers have inherent molecular recognition properties and versatile utilities such as binding ability to small molecules (9), structure-switching function (10,11), and mimics of enzyme function (12). Nucleic acid aptamers have been widely used for small molecule detection due to their high specificities. Since the early 1990s, a variety of small molecules, such as organic dyes (13), amino acids (14,15), nucleotides (16), and various metal ions (17,18), have been explored as potential binding targets of aptamers. With sensitive responsiveness to small molecules as stimuli, the aptamer structure can be reversibly switched by folding into a three-dimensional structure upon binding the target or changing the microenvironment. Several groups have reported a series of structure-switching aptamers with various molecular switches using either modular rational design or *in vitro* selection of communication modules (19–24). These results allow scientists to fuse independently functioning units to multi-functional molecules.

Inspired by these results, we herein report a novel approach: aptamer-mediated control of protein function in response to intracellular small molecules as stimuli. As a model system, we designed a structure-switching aptamer-peptide conjugate (ssAPC) as a kind of hetero-modulator by linking two strand peptides, S10 and S11, derived from tripartite split-GFP (25) to both ends of the ATP binding aptamer that was isolated by structure-switching SELEX (10). The controllability of the aptamer-induced proximity of conjugated S10 and S11 and the subsequent reconstitution of tripartite split-GFP to generate the fluorescence signal was validated using various ATP concentrations *in vitro*. Although aptamer applications *in vitro* with various repertories have been widely accepted, there are extreme hurdles to their practical adoption, even in cells, owing to their easy degradation by nucleases (26). Our aptamer-modulated split GFP platform showed high and long-term stability against nucleases in serum, allowing for its application using intracellular small molecules as environmental stimuli. By transfecting the cells, we demonstrated that our system responds to intracellular ATP levels.

## Materials and methods

### Reagents

Azide-modified split ATP aptamers, splint strands, and inhibitory strands were synthesized and purified by Integrated DNA Technologies (Coralville, IA, USA). DBCO-modified S10 and S11 peptides were synthesized by GenScript (Piscataway, NJ, USA). All sequences are listed in Supplementary Table S1. T4 DNA ligase was purchased from Takara (Kyoto, Japan). ATP was purchased from Thermo Fisher Scientific (Waltham, MA, USA), and GTP, CTP, and UTP were purchased from Promega (Madison, WI, USA). Fetal bovine serum (FBS) was purchased from Thermo Fisher Scientific (Waltham, MA, USA).

### Structure-switching aptamer-peptide complex assembly

Conjugation was performed between the C-terminus DBCO-modified S10 peptide and 5′ azide-modified structure-switching aptamer (5′ SSA), and between N-terminus DBCO-modified S11 peptide and 3′ azide-modified structure-switching aptamer (3′ SSA). To prepare the stock solution, azide-modified DNA and DBCO-modified peptide were dissolved in TE buffer (10 mM Tris and 1 mM EDTA) and DMSO, respectively. Then, 10 μM of DNA and the appropriate concentrations of peptides were diluted using PBS. Split aptamer fragments (10 μM) were then incubated with the DBCO-modified peptide at different ratios ranging from 1:1 to 1:20 in PBS buffer (pH 7.4) overnight at 23°C. All conjugates were analyzed using 10% polyacrylamide gel electrophoresis (PAGE) in TBE (89 mM Tris-borate, 2 mM Na_2_EDTA, pH 8.3) under urea-based denaturing conditions to determine the optimal conjugation ratio without unreacted DNA strands.

The 5′ SSA and 3′ SSA conjugates were then combined and ligated. A 500 μL mixture of 5 μM of the three DNA strands (5′ SSA, 3′ SSA and ACD splint), 1× ligation buffer, and 70 units of T4 DNA ligase were incubated at 37°C overnight. The ligation efficiency was analyzed using PAGE. Finally, the completed aptamer-peptide complex was purified by size-exclusion chromatography using a Superdex 75 10/300 GL column (Cytiva, Marlborough, MA, USA) under UV absorbance monitoring at 254 nm.

### Assessing ATP-mediated reconstitution of tripartite split-GFP

We prepared a 50 μL mixture containing 1 μM aptamer-peptide complex and 20 μM ipGFP1-9 in ATP binding buffer (ATPBB; 20 mM Tris, 300 mM NaCl and 5 mM MgCl_2_, pH 7.4). These mixtures were transferred to a 384-well black microplate (Greiner Bio One, Kremsmünster, Austria) and challenged with various concentrations of ATP (2–10 mM). Fluorescence intensities were measured over a time course of 20 h at 5 min intervals using the extended mode of Cytation 7 (BioTek, Winooski, VT, USA) with excitation/emission wavelengths of 488/525 nm.

### Mouse serum collection for long-term stability experiments

Whole blood was collected from C57BL/6N mice by terminal cardiac puncture after anesthetization with an intraperitoneal injection of avertin. The collected whole blood was allowed to clot at 23°C for 20 min, and then centrifuged at 8000 × g for 15 min at 4°C to collect the serum.

### Cell culture and imaging experiments

A549 human lung carcinoma cells were obtained from the Korean Cell Line Bank (Seoul, South Korea). The cells were cultured at 37°C and 5% CO_2_ in DMEM containing 10% FBS (v/v) and penicillin/streptomycin (Thermo Fisher Scientific, Waltham, MA, USA). A549 cells were seeded at a density of 1 × 10^4^ cells/well in 96-well cell culture black plates (Greiner Bio One, Kremsmünster, Austria). After 24 h, the cells were transfected with naïve GFP, ipGFP1-9, and aptamer-peptide complexes using Lipofectamine 3000 (Thermo Fisher Scientific, Waltham, MA, USA) according to the manufacturer's instructions. The cells were imaged using the Cytation 7 Cell Imaging Multi-Mode Reader (BioTek Instruments, Winooski, VT, USA) with excitation/emission wavelengths of 488/525 nm for 24 h under cultivation conditions with 5% CO_2_ at 37°C. Images were analyzed using ImageJ software (https://imagej.nih.gov).

For starvation experiments, A549 cells were seeded at a density of 1 × 10^6^ cells/dish in 100 mm^2^ cell culture dishes. After incubation for 24 h, the medium was changed to DMEM without glucose. The cells were harvested and stored at –80°C until the ATP assay was performed. Intracellular ATP concentrations were measured using an ATP assay kit (Abcam, Cambridge, MA, USA) according to the manufacturer's instructions.

### Statistical analysis

All experiments were performed at least three times. The data are presented as mean ± standard deviation (SD). Statistical analyses were performed using a *t*-test and *P*-values less than 0.05 were considered statistically significant.

## Results and discussion

### Design of aptamer-split peptide conjugate as hetero-modulator for GFP

**Figure 1.**
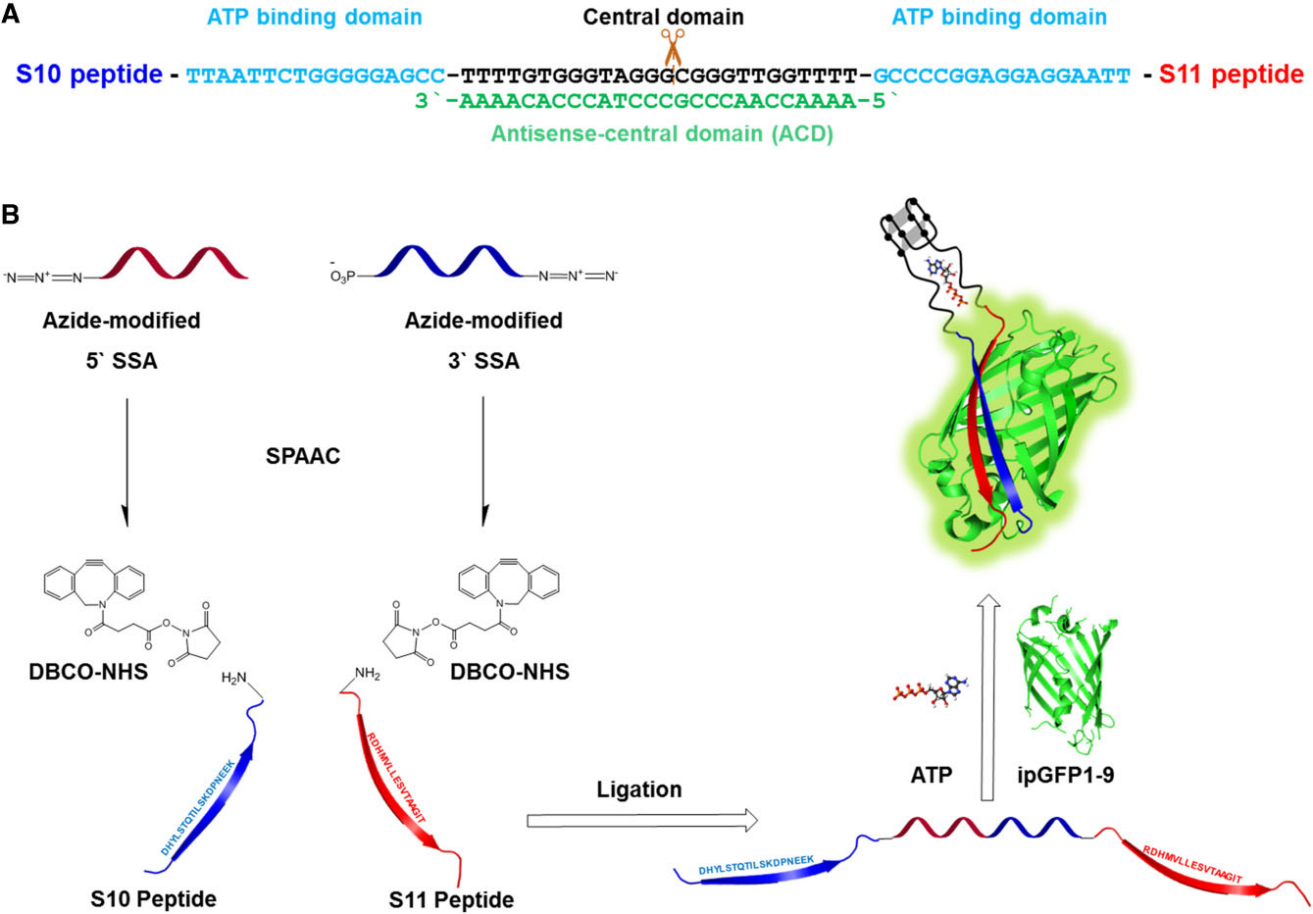
Schematic of the aptamer-modulated split GFP design and assembly process. (**A**) Sequence of the structure-switching ATP binding aptamer with conjugated S10 and S11 peptides. The ATP-binding domain, is marked in light blue; the central domain, marked in black; and the splint sequence for ligation, marked in green. (**B**) Strategy for aptamer-peptide conjugation by click chemistry and the reconstitution of tripartite split-GFP by the structure-switching response of the aptamer in the presence of ATP.

To construct an aptamer-based modulator for split GFP, we prepared a divided version of an existing structure-switching ATP-binding aptamer (10) (Figure 1A). This sequence was discovered by structure-switching SELEX using a library comprising molecules in which a conserved 26-nucleotide (nt) central domain is flanked by two randomized 17-nt sequence domains that participate in target binding. The aptamer was divided into two fragments, referred to as 5′ SSA and 3′ SSA, with the central domain as the split site (see Supplementary Table S1 for details). These two aptamer fragments were then coupled to two split peptides from a tripartite split-GFP construct (S10 and S11) using the strain-promoted azide-alkyne cycloaddition (SPAAC) reaction, wherein azide groups at the 5′ end of 5′ SSA and 3′ end of 3′ SSA reacted with DBCO functional groups coupled to the C-terminus of S10 and N-terminus of S11 (Figure 1B). To complete the structure-switching aptamer-peptide conjugate (ssAPC), these two constructs were joined together via a ligation reaction. In this design, we considered the direction from the N- to C-terminus of the GFP folding structure, characterized by a beta-barrel structure with eleven beta-strands surrounding chromophores. This strategic alignment ensures compatibility with the natural folding pathway of GFP (25). We strategically inserted the ATP aptamer into the loop region between the S10 and S11 peptide to facilitate efficient reassembly upon aptamer binding with a target molecule. The remaining GFP component, GFP1-9, produces minimal fluorescence until the other two peptides were brought into close proximity by specific interactions. In the presence of ATP, the aptamer becomes fully-folded, bringing the S10 and S11 peptide moieties together, enabling subsequent assembly of the full tripartite split-GFP complex with GFP1-9, producing a strong fluorescent signal. Unlike other split-protein systems such as split-luciferase and split-DHFR, tripartite split-GFP does not require any substrate to produce fluorescent signal and also has a lower false-positive rate than bipartite split-GFP systems.

First, we optimized the molar ratios for the aptamer-peptide conjugation step for the SPAAC reaction. Azide-modified split ATP aptamers were conjugated with various ratios of DBCO-modified peptides (ranging from 1:1 to 1:20) using SPAAC to prepare aptamer-peptide conjugates. After overnight incubation, the azide-modified 5′ SSA aptamer fragment was completely conjugated with the C-terminus DBCO-modified S10 peptide when combined at 1:20, whereas 1:5 was required for efficient conjugation of the azide-modified 3′ SSA with the N-terminus DBCO-modified S11 peptide (Supplementary Figure S1). As shown in Figure 1A, we employed a 26-nt antisense-central domain (ACD) strand as a splint for ligation between the two segments of the ATP aptamer. The ACD stabilizes the double-stranded complex to avoid self-folding, which can cause false-positive signals in the absence of ATP binding. In the presence of ATP, the ACD strand was displaced by the ligand (ATP), allowing the aptamer to fold properly. A previous study investigated the concentration of ATP required to achieve release from this aptamer for antisense strands of varying lengths (27). We initially attempted to use short strands to increase aptamer sensitivity by reducing the strength of ACD hybridization, but the resulting lower melting temperature adversely influenced ligation efficiency. PAGE analysis under urea-based denaturing conditions confirmed the higher ligation efficiency using the 26-nt ACD strand as a splint (Supplementary Figure S2). Finally, the completed ssAPC was purified by size-exclusion chromatography so that the remaining ligase and buffer components could be removed while keeping the ACD strand hybridized to the aptamer-peptide construct (Supplementary Figure S3).

### *In vitro* validation of aptamer-split peptide hetero-modulator for GFP

**Figure 2.**
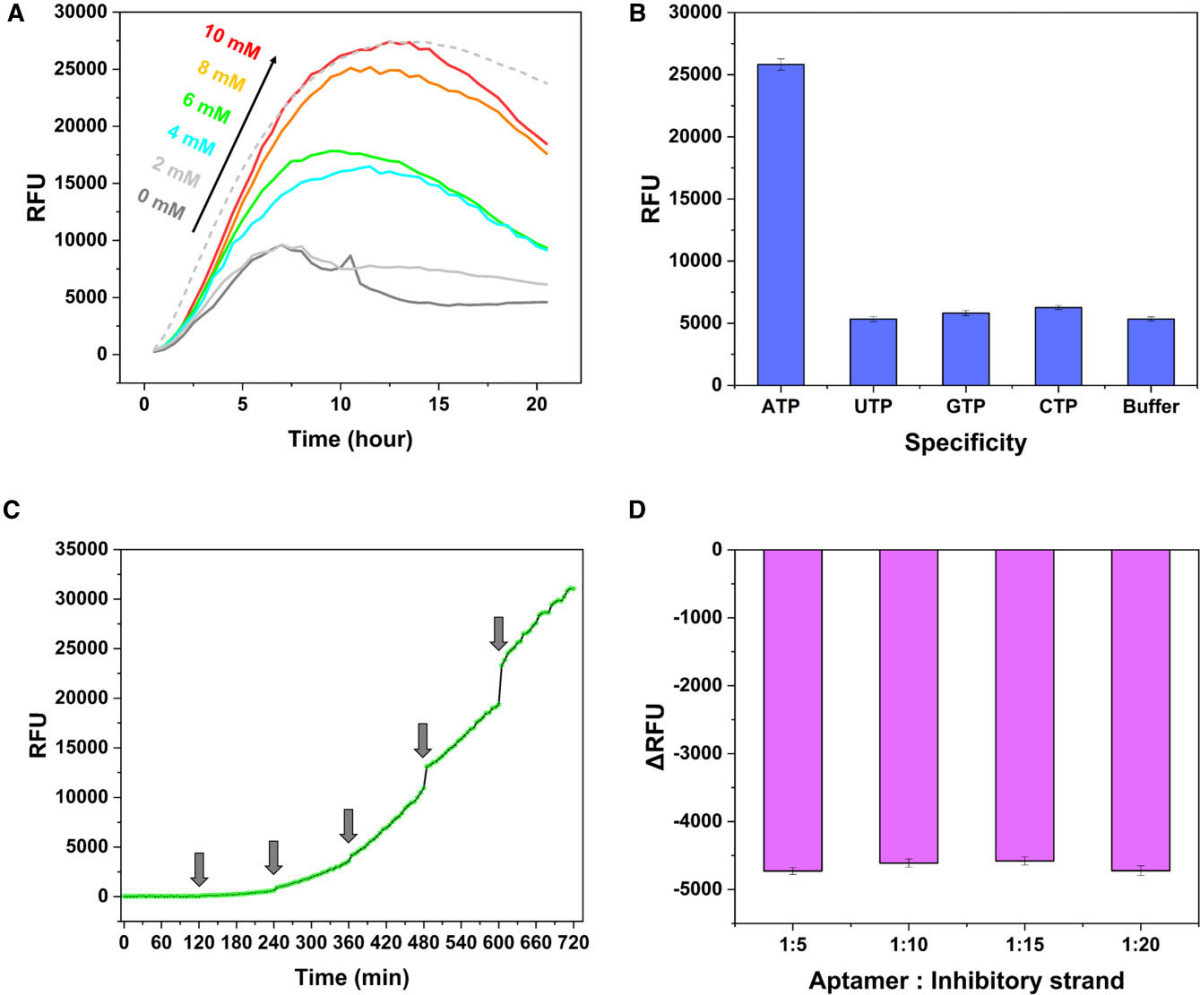
Validation of the performance and specificity of aptamer-split peptide hetero-modulator for GFP. (**A**) Time-course of ssAPC/ipGFP1-9 assembly and fluorescent signaling in response to various concentrations of ATP. The light gray dash line indicated positive control using reassembly of ipGFP1-9 with synthesized S10-11 peptide. (**B**) Specificity of ssAPC/ipGFP1-9 assembly in the presence of 10 mM ATP, GTP, CTP, and UTP, respectively. Fluorescence intensities were compared after incubation for 12 h. (**C**) Monitoring the sustained response of aptamer-modulated split GFP with stepwise increases in ATP concentration. Arrows indicate the addition of 2 mM ATP every two hours. (**D**) Assessment of the importance of structure-switching for aptamer-modulated split GFP response. We incubated the construct with 10 mM ATP for 12 h before adding different ratios of an inhibitory strand that was fully complementary to the aptamer sequence and thus impedes target-induced folding. Individual fluorescence before and after the addition of the inhibitory strand was measured, and changes in relative fluorescence intensity were calculated. Error bars indicate standard deviations of fluorescence intensities evaluated at multiple time points.

To assess the functionality of the aptamer-split peptide conjugate for GFP function modulation, we performed a time-course *in vitro* reconstitution assay with ssAPC and split GFP1-9 in the presence of varying concentrations of ATP (Figure 2A). Our group previously reported an *in vitro* reconstitution application using intein-mediated purified split GFP1-9 (ipGFP1-9) with improved solubility and expression levels to provide evidence of compatibility for application in this study (28). A previous study demonstrated the reconstitution between S10-11 linked peptide and ipGFP1-9 as a positive control with a time-course fluorescent readout (Figure 2A, light gray dash line, and Supplementary Figure S4), and we observed similar result in this aptamer-modulated split GFP system. We showed that high concentrations of ATP could trigger the full assembly of tripartite split-GFP molecules, generating a measurable fluorescent readout. In the absence of ATP, we observed a modest background signal that could yield a false-positive result at earlier time-points (Figure 2A, dark gray trace), although this could readily be distinguished as a negative result relative to ATP-containing samples at later time-points. ATP concentrations ≥4 mM proved sufficient to generate a measurable positive response from our system, whereas the 2 mM ATP sample was largely indistinguishable from the negative control. As aforementioned, according to Kang *et al.*, the performance of this ATP aptamer has been proven by measuring EC_50_ values in terms of ATP-induced structure-switching with different lengths of antisense DNAs (11–18 nt) (27). A shorter, 11-nt antisense DNA showed a lower EC_50_ of 238 μM, whereas a much longer antisense strand (18-nt) produced a >20-fold higher EC_50_ value of 4768 μM owing to its hybridization energies.

The specificity of this ATP aptamer has been demonstrated in previous reports (10,27). We also verified the specificity of our system towards ATP as a trigger molecule by assessing its response to other nucleotides such as UTP, GTP and CTP under identical conditions. To validate the low false-positive rate of self-assembly or non-specific binding, we compared the recovered fluorescence intensities after 12 h of incubation with 10 mM of each nucleotide. Fluorescence recovery was highly ATP-specific, with a minimal response observed with other nucleotides (Figure 2B).

We subsequently evaluated the performance of our system in the context of sustained responsiveness to environmental ATP changes. We conducted an experiment in which we measured fluorescence intensity over time while adding extra ATP in 2 mM increments every 120 min, up to a final concentration of 10 mM. We observed that the fluorescence intensity increased monotonically, and the intensities jumped in a stepwise manner when extra ATP was added (Figure 2C). These data demonstrate that our aptamer-modulated system could sophisticatedly control protein function by changing microenvironmental stimuli.

To verify that the fluorescence signal resulted from ATP-induced structure-switching, we performed experiments with the aptamer-modulated split GFP, in which we included an inhibitory strand that was fully complementary to the ATP aptamer sequence. We first performed an assembly reaction with the ssAPC, ipGFP1-9, and ATP for 12 h to generate a strong fluorescent signal, after which we added the inhibitory strand at varying molar ratios relative to the ssAPC ranging from 1:5 to 1:20 (Figure 2D). Under all these conditions, we observed a strong decrease in fluorescence in the presence of the inhibitory strand, confirming that ATP-induced folding of the aptamer is essential to generate a fluorescent readout.

### Assessing the nuclease-resistance of the aptamer-split peptide hetero-modulator

As mentioned above, DNA aptamers have extreme hurdles for use *in vivo* because of their weak stability against DNA nuclease in serum (26). To overcome this limitation, numerous chemical modifications at isolated aptamers have been attempted (29,30). However, these modifications also have drawbacks. In terms of pre-SELEX modification, the preparation cost of a random library can be increased, and the polymerase type has limitations in amplifying modified nucleotides. In the case of post-SELEX modification, the folding structure of the isolated aptamers could be affected, resulting in difficulties in sustaining the binding affinity and specificity of the target molecule. Therefore, the need to improve the *in vivo* stability of nucleic acid aptamers without pre- or post-SELEX modifications still remains.

**Figure 3.**
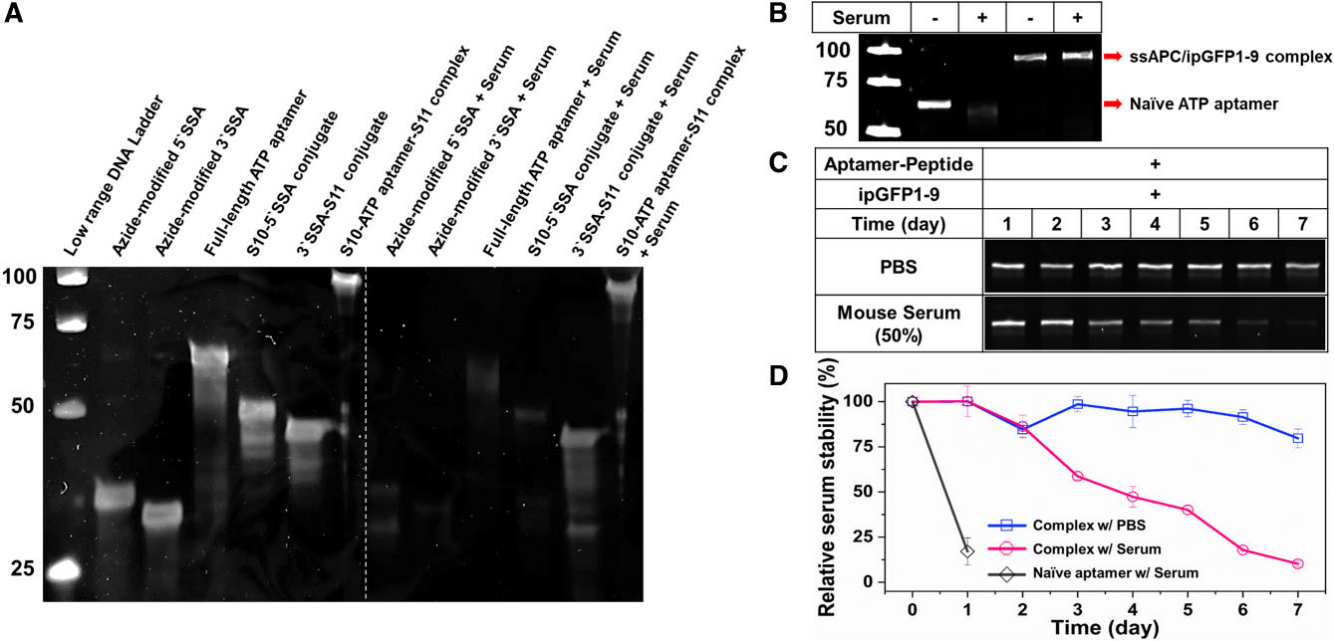
Stability of aptamer components and aptamer-split peptide hetero-modulator against nuclease degradation. (**A**) Samples were incubated with 10% fetal bovine serum (FBS) at 37°C for 16 h, and unreacted naïve aptamers were used as controls. (**B**) Stability of reconstituted ssAPC/ipGFP1-9 complex in fresh mouse serum. All samples were subjected to urea-denaturing polyacrylamide gel electrophoresis (PAGE) analysis. For size determination, we used a 25-bp low-range DNA ladder. (**C**) Long-term stability in fresh mouse serum. The ssAPC/ipGFP1-9 complex was incubated with 50% fresh mouse serum or PBS. After sampling every 24 h for 7 days, all samples were analyzed using denaturing PAGE, and (**D**) band intensities were quantified by densitometry of the scanned gel images using ImageJ software.

Our approach has high stability against exonucleases due to the conjugation of two peptides at both ends of the aptamer and is protected against endonucleases when the ssAPC is fully reconstituted with ipGFP1-9. To demonstrate this nuclease resistance, we carried out a stability test of both the unmodified aptamers and the peptide-conjugated construct (ssAPC) by incubating in 10% fetal bovine serum (FBS) at 37°C for 16 h. Unreacted naïve aptamers with FBS were used as the controls. Analysis by urea-denaturing PAGE showed that the split aptamer components and full-length aptamer were completely degraded by the nucleases present in 10% FBS. In contrast, the peptide-conjugated construct (ssAPC) exhibited only partial degradation, indicating significantly higher resistance to nuclease attack (Figure 3A). We further tested the stability of the reconstituted ssAPC/ipGFP1-9 complex in a more aggressive serum stability assay, using 50% fresh mouse serum at 37°C for 24 h (Figure 3B). The reconstituted complex demonstrated higher stability than the partially degraded un-reassembled ssAPC, reinforcing the resistance to nucleases. These results indicate that our ssAPC construct is durable against rapid nuclease-mediated degradation.

To evaluate the long-term nuclease resistance of the aptamer-split peptide hetero-modulator, we conducted a serum stability assay using 50% diluted fresh mouse serum at 37°C over 7 days. Samples were collected every 24 h to monitor degradation. The results indicated that the aptamer-split peptide hetero-modulator demonstrated robust long-term stability, with a half-life of approximately four days (Figure 3C and D). Despite the high nuclease activity in the serum, the construct maintained its integrity for several days, highlighting its resistance to nuclease-mediated degradation. These data indicate that our approach exhibits excellent serum stability for an extended period, and is suitable for long-term *in vivo* applications in response to intracellular small molecules. Compared with other chemically modified aptamers exhibited around 12–24 h of half-life in the serum (31), our system has significantly robust stability and no need for backbone/base modifications of the aptamer structure.

To date, FRET-based aptamer biosensors have been typically employed either a fluorophore-quencher pair or two fluorophores positioned at specific sites on an aptamer. As the aptamer undergoes conformational changes upon binding to a target molecule, the distance between the fluorophores changes, resulting in a measurable fluorescence signal. These systems are valued for their rapid response times, often within seconds to a few minutes, making them highly suitable for real-time monitoring of molecular interactions (32). However, this rapidity can pose challenges, including a higher risk of false positives if the fluorophore separates from the quencher due to aptamer degradation. In contrast, the aptamer-split peptide hetero-modulator relies on the reassembly of tripartite split-GFP components, initiated by a structure-switching aptamer. This mechanism is slower than FRET-based systems, as it requires complete reassembly of the split GFP fragments to generate a fluorescence signal responding to the increased stimuli. While the aptamer-ATP reaction is rapid, the split GFP reassembly is slow, and once reconstituted, it is challenging to reverse. Despite the slower kinetics, this system offers notable advantages, including reduced false positives and greater stability against nuclease degradation. The complete reassembly of split GFP mitigates the risk of unintended fluorescence, making it suitable and reliable under complex biological environments. These features make the aptamer-split peptide hetero-modulator a more robust system compared to FRET-based SSAs, particularly in applications where stability and low false-positive rates are crucial. The enhanced stability and resistance to degradation are advantageous for various applications, including research and diagnostics for intracellular compound levels, and potential therapeutic strategies.

### Validation of aptamer-modulated split GFP responsiveness to intracellular ATP

**Figure 4.**
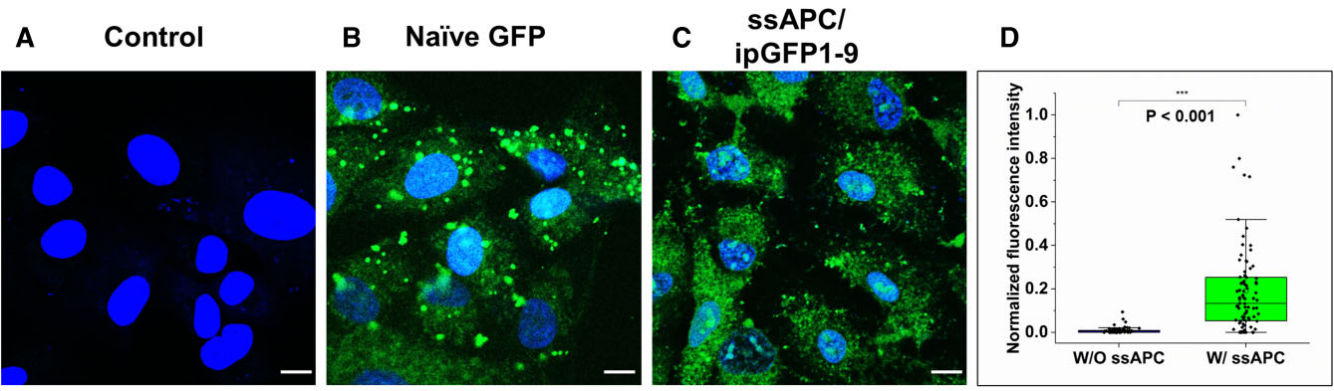
Assessment of the response of aptamer-modulated split GFP to intracellular ATP in live A549 cells. Images of A549 cells with (**A**) untransfection as control, (**B**) naïve GFP, and (**C**) ssAPC/ipGFP1-9. Scale bar = 10 μm. The figure shows representative data of three independent experiments. (**D**) Box plots depicting fluorescence intensities of ssAPC/ipGFP1-9 transfected A549 cells. Box, 25^th^–75^th^ percentile; solid line, median; T-bars, 5^th^–95^th^ percentile; *n* = 83. Images were quantified using ImageJ software (*n*= 3–5 images of each group).

Finally, we assessed the functionality of aptamer-modulated split GFP to respond to intracellular ATP levels via the reassembly within cells. Healthy cells typically have intracellular ATP concentrations ranging from 1 to 10 mM, whereas cancer cells contain much higher levels due to increased glycolysis during tumor proliferation and angiogenesis (33). Given that our aptamer-modulated split GFP system can readily discriminate ATP concentrations ≥4 mM and has long-term serum stability, we anticipated that it would be well-suited for responding to endogenous intracellular ATP concentrations. Following the approach for the intracellular delivery of exogenous proteins (34), we used Lipofectamine to transfect ssAPC and ipGFP1-9 together (Figure 4), naïve GFP as a positive control (Figure 4B), or ipGFP1-9 alone as a negative control (Supplementary Figure S5) into the A549 human lung cancer cell line. We observed a robust fluorescent signal from the ssAPC/ipGFP1-9-transfected cells (Figure 4C), indicating that ssAPC was successfully reconstituted with ipGFP1-9 to generate a readout in response to intracellular ATP.

In addition, we examined the human bronchial epithelial cell line BEAS-2B as a comparator (Supplementary Figure S6). Following the incubation with ssAPC/ipGFP1-9, our comparative analysis revealed that the fluorescence intensity in the A549 cell line was significantly higher than that in the BEAS-2B cell line. This finding indicates a relatively higher ATP level in the A549 cells compared to the BEAS-2B, validating our experimental approach and supporting our conclusions. These findings suggest that ssAPC/ipGFP1-9 can differentiate between cell types based on intracellular ATP levels, indicating that this approach could be valuable in developing cancer diagnostics or potentially serving as a therapeutic agent. The ability to discern ATP levels in different cell types, especially between normal and cancerous cells, offers a promising pathway for future applications in cancer research and treatment strategies.

**Figure 5.**
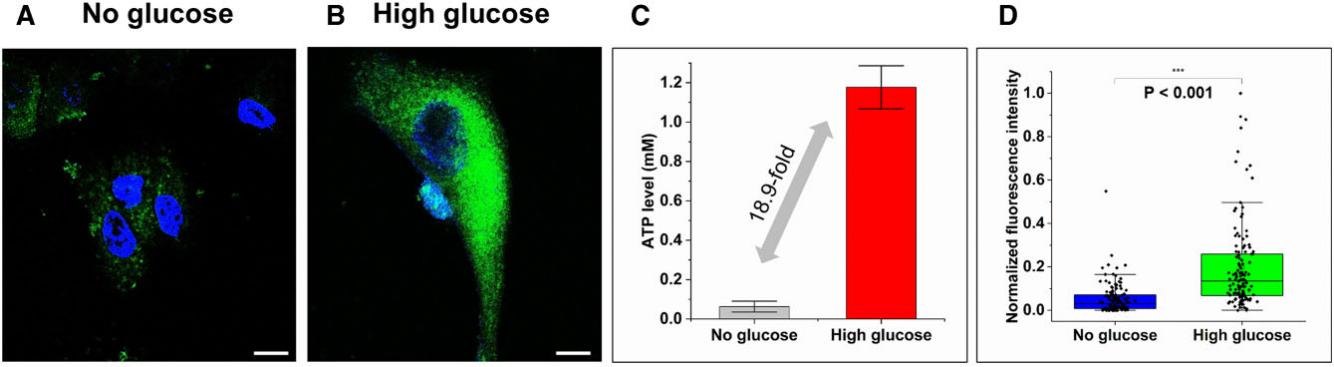
Proving the aptamer-modulated split GFP to respond to intracellular ATP using cell culture-induced ATP changes. ssAPC and ipGFP1-9 were transfected into (**A**) cultured A549 cells in a glucose-free medium and (**B**) cultured A549 cells in a high-glucose medium using Lipofectamine. The scale bar indicates 10 μm. The figure shows representative data of 3 independent experiments. (**C**) ATP levels were determined in A549 cells cultured in a medium with or without glucose. (**D**) Fluorescence intensities were quantified from the cell images using ImageJ software (*n*= 3–5 images of each group). Box, 25^th^–75^th^ percentile; solid line, median; T-bars, 5^th^–95^th^ percentile; white square, mean; *n* = 128.

We subsequently introduced perturbations to this cell culture model to investigate how marked changes in intracellular ATP levels influenced the fluorescence signal produced by the developed system. Cellular ATP levels are directly related to glucose levels in the culture medium; therefore, we cultured A549 cells in glucose-free medium to see how the signal changes in response to the resulting decrease in ATP levels (Figure 5 and Supplementary Figure S7). We confirmed the ATP levels of both A549 lung cancer cell lines using a commercial ATP assay kit (Figure 5C), followed by transfection with the cells under identical conditions. As expected, this treatment resulted in the loss of green fluorescence signal as a consequence of glucose concentrations dropping below detectable levels. Additionally, we conducted an ATP depletion assay to strengthen our results (Supplementary Figure S8). We used Oligomycin A, an ATP synthase inhibitor that impedes the mitochondrial proton channel, to induce ATP depletion (35). These data demonstrate that our aptamer-modulated split GFP has robust stability within cells and that this system is controlled by sensitive and selective responses to intracellular ATP levels.

## Conclusion

In this study, we designed an aptamer-peptide conjugate as a modulator for split proteins, which is a novel type of DNA-mediated control of protein functions in response to small-sized trigger molecules. Specifically, by linking S10 and S11 peptides derived from the tripartite split-GFP to both ends of the structure-switchable ATP aptamer, we created a structure-switching aptamer-peptide conjugate (ssAPC) as a hetero-modulator to enable GFP to respond to the presence of ATP. We demonstrated that the reconstitution reaction occurred via conformational changes in the aptamer when ATP was present *in vitro*. Our design provides several advantages over other systems, which exhibited lower false-positive rates and high signal-to-noise compared to fluorescence/quencher pair systems or FRET-based strategies, enhancing its reliability in complex cellular environments. Furthermore, our hetero-modulator provides high stability against nuclease degradation within cells and long-term stability with a half-life of approximately four days. Finally, we demonstrated that the reconstitution of split GFP was successfully controlled only by the responsiveness of the designed DNA–peptide hetero-modulator to intracellular ATP levels.

Despite the millimolar levels of high concentration to stimuli required and its slower kinetics due to the reassembly process, the ssAPC/ipGFP1-9 system offers unique benefits, including the ability to replace the aptamer sequence to target different small molecules. This flexibility can broaden the range of applications for this platform, allowing for diverse metabolite monitoring uses. Ultimately, we showed that the reconstitution of split GFP was successfully controlled by the DNA–peptide hetero-modulator's responsiveness to intracellular ATP levels, suggesting a promising pathway for future biosensor development.

## Supplementary Material

gkae532_Supplemental_File

## Data Availability

The data underlying this article are available in the article and in its online supplementary data.
