# Supplementary material for: DNA-controlled protein fluorescence: Design of aptamer-split peptide hetero-modulator for GFP to respond to intracellular ATP levels
Source: Nucleic Acids Res. 2024 Jun 25;52(14):8063–71. doi: 10.1093/nar/gkae532 (PMC11317148; doi:10.1093/nar/gkae532)
Supplement: gkae532_Supplemental_File [file gkae532_supplemental_file.docx]

*Supplementary Information*

DNA-Controlled Protein Fluorescence: Design of Aptamer-Split Peptide Hetero-Modulator for GFP to Respond to Intracellular ATP Levels

Ki Sung Park^1,3,†^, Hanvit Cha^2,3, †^, Jia Niu^4^, Hyongsok Tom Soh^5,6^, Jin Hyup Lee^2^*, and Seung Pil Pack^1^*

1. Department of Biotechnology and Bioinformatics, Korea University, Sejong 30019, Republic of Korea,

2. Department of Food and Biotechnology, Korea University, Sejong 30019, Republic of Korea,

3. Biological Clock-based Anti-Aging Convergence RLRC, Korea University, Sejong 30019, Republic of Korea,

4. Department of Chemistry, Boston College, Chestnut Hill, Massachusetts, 02467, United States,

5. Department of Electrical Engineering, Stanford University, Stanford, CA 94305, United States,

6. Department of Radiology, Stanford University, Stanford, CA 94305, United States

†These authors contributed equally.

*Corresponding author:

Jin Hyup Lee - Email: [jinhyuplee@korea.ac.kr](mailto:jinhyuplee@korea.ac.kr)

Seung Pil Pack - Email: [spack@korea.ac.kr](mailto:spack@korea.ac.kr)

**Contents**

**Table S1.** DNA aptamer and peptide sequences used in this study.

**Figure S1.** Optimization of the molar ratios between the azide-modified aptamer and DBCO-modified peptide for aptamer-peptide conjugation using click chemistry.

**Figure S2.** Assessing the aptamer-peptide conjugates ligation reaction.

**Figure S3.** Purification of completed aptamer-peptide complexes by size-exclusion chromatography after ligation.

**Figure S4.** Reconstitution of tripartite split GFP with synthesized peptides.

**Figure S5.** Response of aptamer-modulated split GFP to intracellular ATP in live A549 cells.

**Figure S6.** Comparison of the ability to distinguish cell types between normal and cancer cells using the aptamer-modulated split GFP system.

**Figure S7.** Validation of aptamer-modulated split GFP response to intracellular ATP using cell culture-induced ATP changes.

**Figure S8.** ATP depletion assay using Oligomycin A.

**Table S1.** DNA aptamer and peptide sequences used in this study

| Name | Sequence  (5` to 3` and N to C terminus) | Description |
| --- | --- | --- |
| 5` SSA  (31 mer) | N3-TTAATTCTGGGGGAGCCTTTTGTGGGTAGGG | Split ATP aptamer, Azide modification at 5` end |
| 3` SSA  (29 mer) | PO3-CGGGTTGGTTTTGCCCCGGAGGAGGAATT-N3 | Split ATP aptamer, Phosphorylation modification at 5` end and Azide modification at 3` end |
| Splint strand + poly T linker (12 mer + 10 mer) | AACCCGCCCTAC | Complementary sequence with a central domain for ligation between 5` SSA and 3` SSA  The linker was designed for the purification step in the downstream applications |
| Splint strand + poly A linker (26 mer + 10 mer) | AAAACCAACCCGCCCTACCCACAAAA |  |
| Inhibitory strand (60 mer) | AATTCCTCCTCCGGGGCAAAACCAACCCGCCCTACCCACAAAAGGCTCCCCCAGAATTAA | Full-length complementary strand of ATP aptamer for binding inhibition |
| S10 peptide | DHYLSTQTILSKDPNEEK -DBCO | S10 strand for reconstitution, DBCO modification at C-terminal end for conjugation with 5` SSA |
| S11 peptide | DBCO-RDHMVLLESVTAAGIT | S11 strand for reconstitution, DBCO modification at N terminal end for conjugation with 3` SSA |


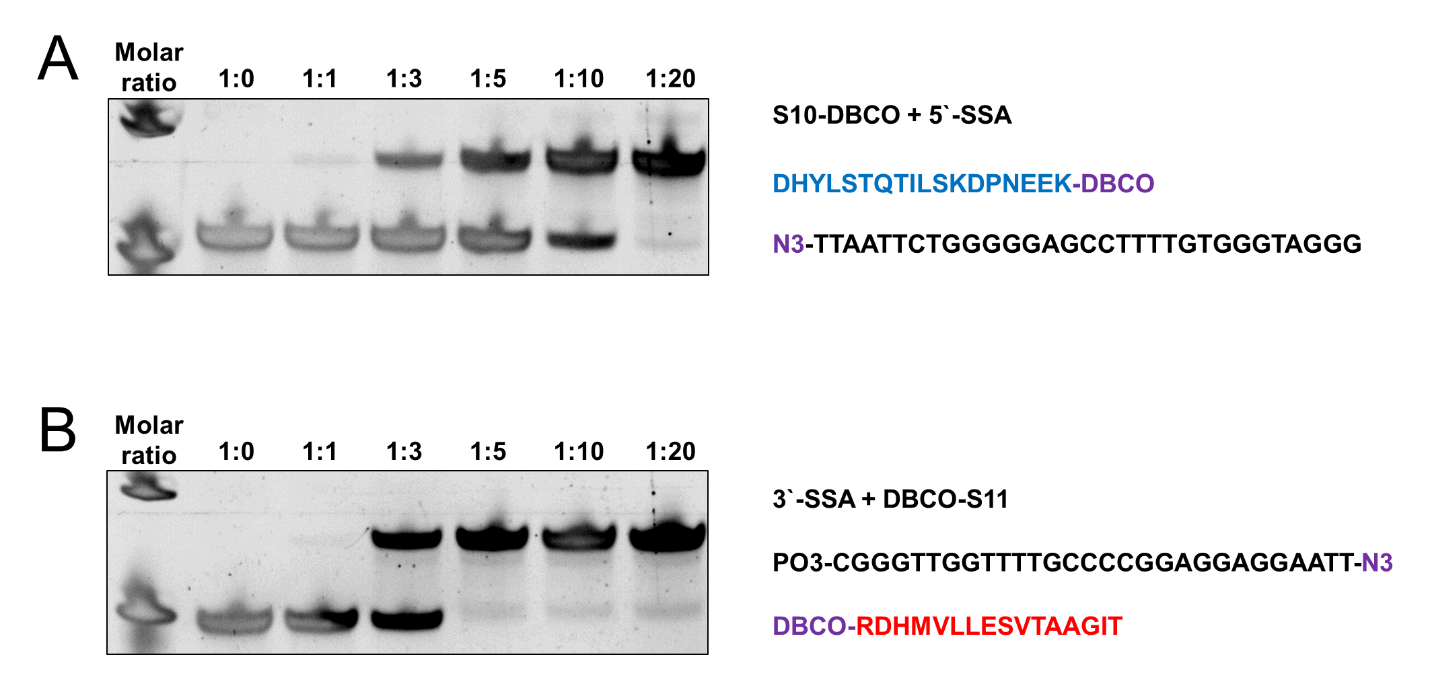


**Figure S1.** Optimization of the molar ratios between the azide-modified aptamer and DBCO-modified peptide for aptamer-peptide conjugation using click chemistry. 10 µM of azide-modified split ATP aptamer fragments were reacted with DBCO-modified peptides at various ratios (1:0, 1:1, 1:3, 1:5, 1:10, and 1:20).


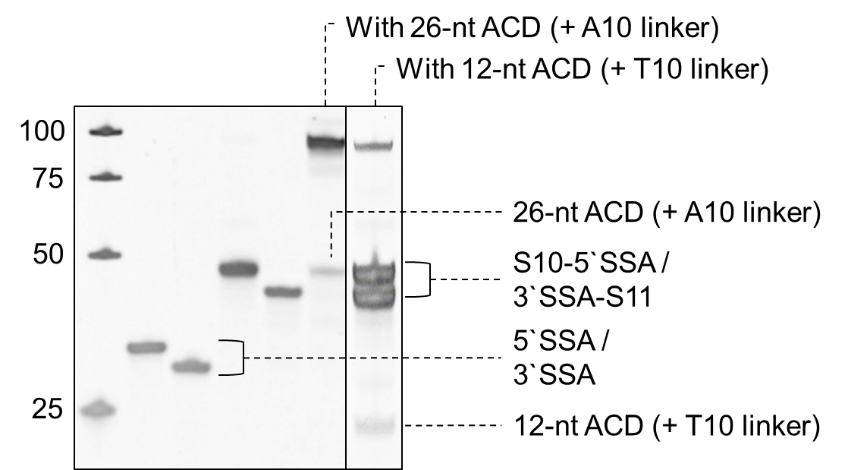


**Figure S2.** Assessing the aptamer-peptide conjugates ligation reaction. Lane 1, 25 bp low-range DNA ladder; lane 2, azide-modified 5’SSA; lane 3, azide-modified and phosphorylated 3’SSA; lane 4, S10 peptide-conjugated 5’SSA; lane 5, S11 peptide-conjugated 3’SSA; lane 6, ligation product between S10-5’SSA conjugated and 3’SSA-S11 conjugate using 26-nt ACD as a splint; lane 7, ligation product between S10-5’SSA conjugated and 3’SSA-S11 conjugate using 12-nt ACD as a splint. The linker was designed for the purification step in the downstream application. All samples were loaded onto a 10% TBE-urea-denaturing polyacrylamide gel electrophoresis (PAGE) and run at < 60°C.


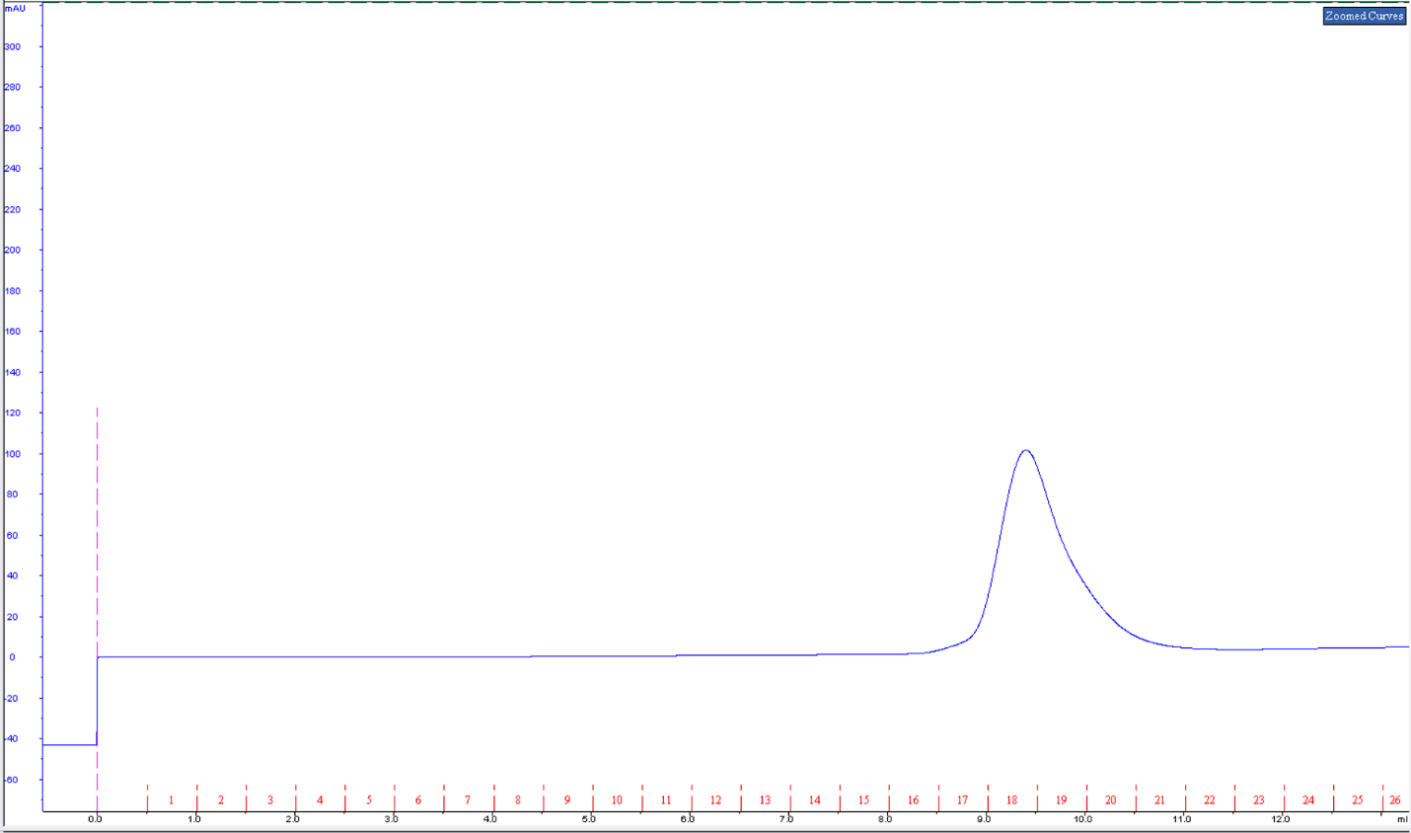


**Figure S3.** Purification of completed aptamer-peptide complexes by size-exclusion chromatography after ligation. UV absorbance was monitored at 254 nm to detect absorption by oligonucleotides.


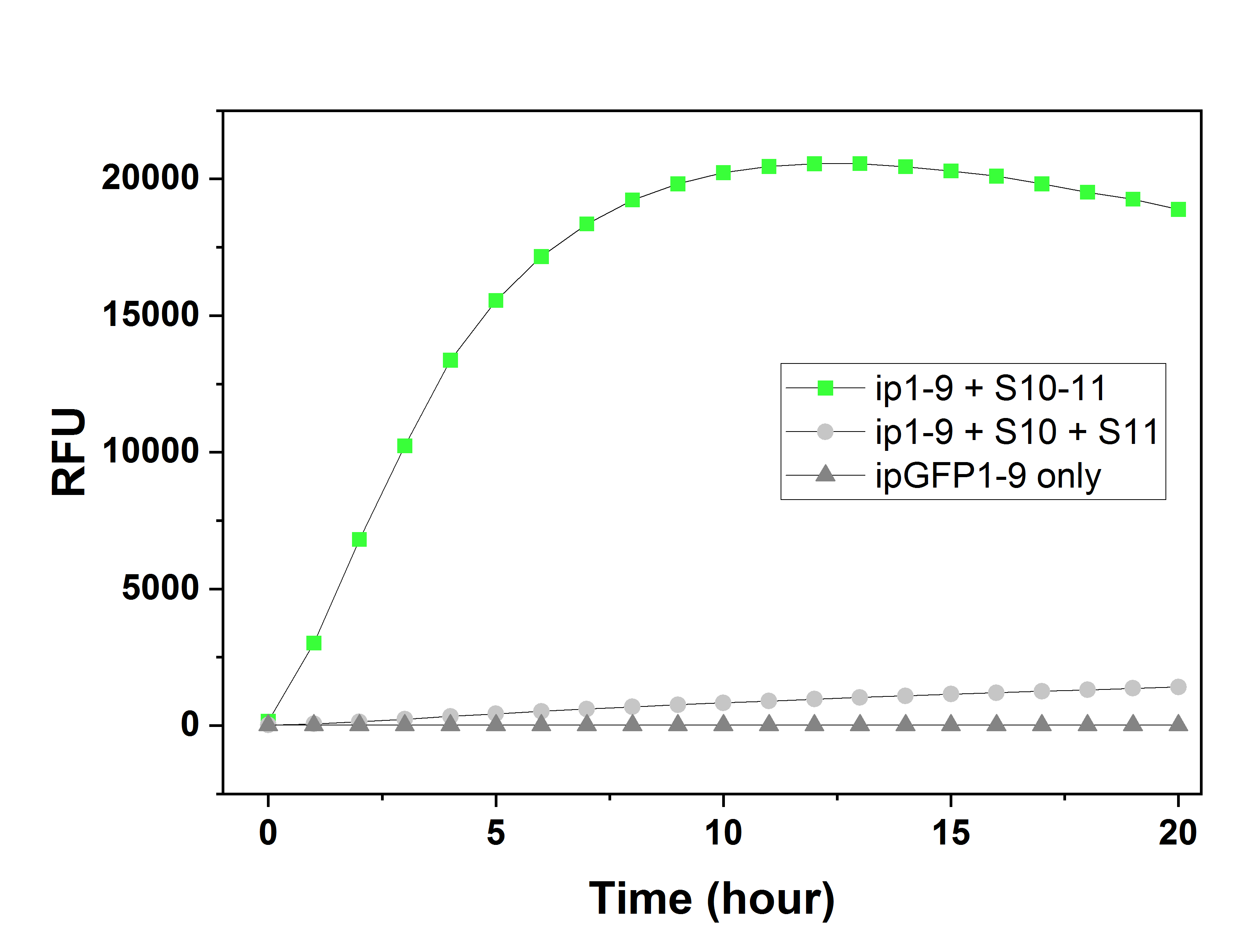


**Figure S4.** Reconstitution of tripartite split GFP with synthesized peptides. Time-course reconstitution was performed using ipGFP1-9 alone or with a S10-11 linked peptide or the separate S10 and S11 peptides.


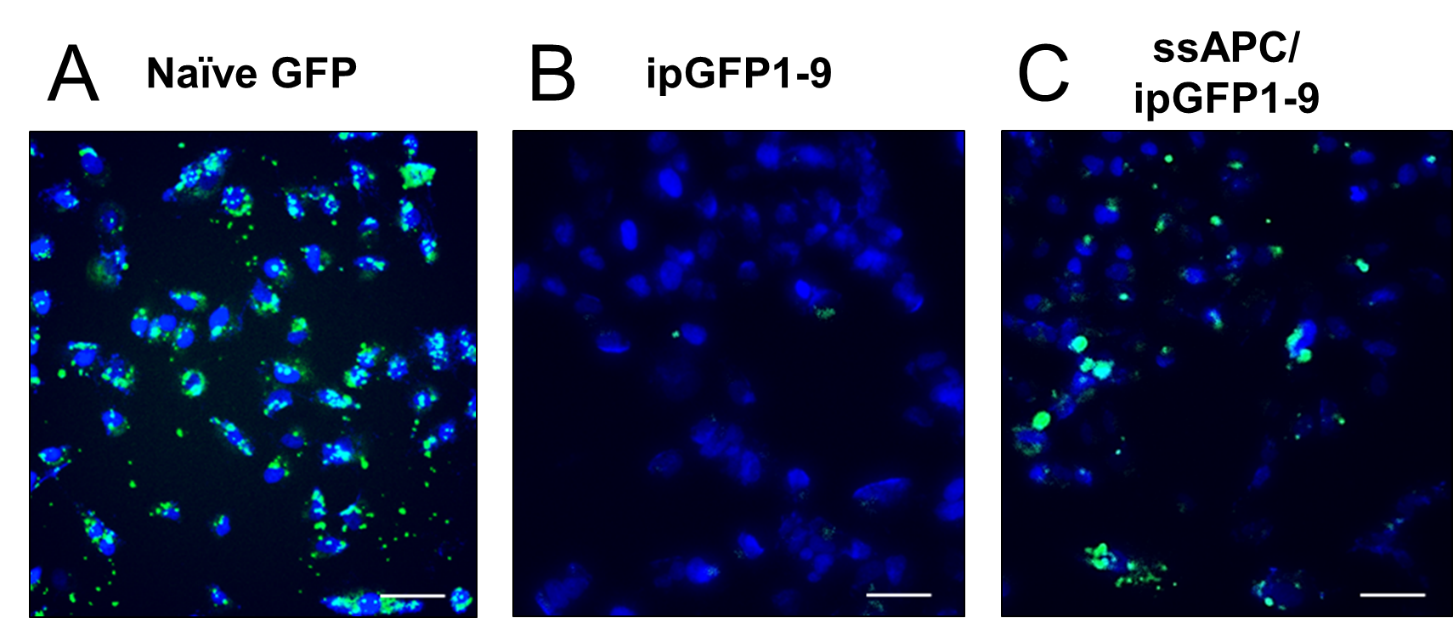


**Figure S5.** Response of aptamer-modulated split GFP to intracellular ATP in live A549 cells. Images of A549 cells with (A) naïve GFP, (B) ipGFP1-9 only, and (C) ssAPC/ipGFP1-9. Scale bar = 50 µm. The figure shows representative data of 3 independent experiments.

**
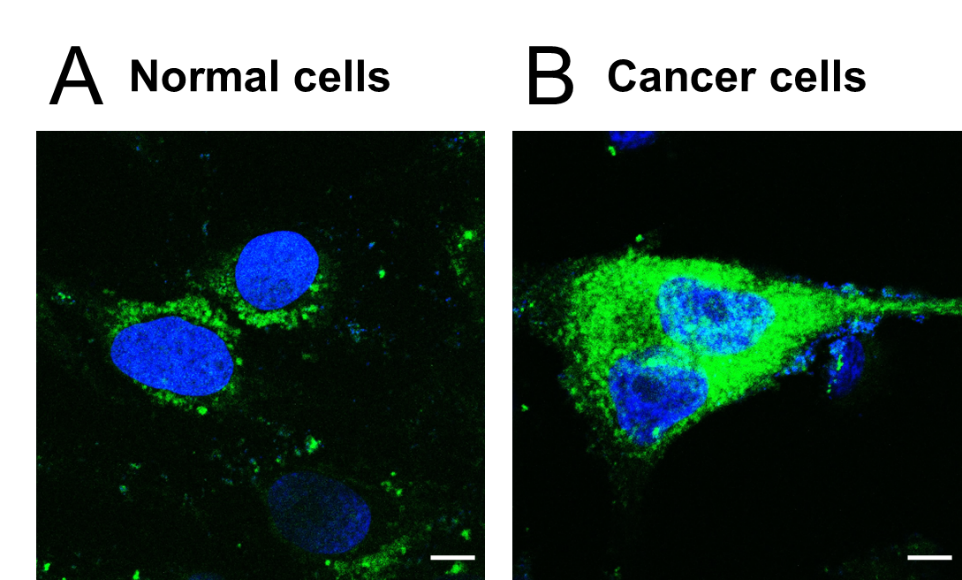
**

**Figure S6.** Comparison of the ability to distinguish cell types between normal and cancer cells using the aptamer-modulated split GFP system. The bronchial epithelial cell line BEAS-2B (representing normal cells) and the lung adenocarcinoma cell line A549 (representing cancer cells) were used for comparison. The fluorescence intensity represents the relative ATP levels within each cell type. The scale bar indicates 10 µm. The figure shows representative data from 3 independent experiments.


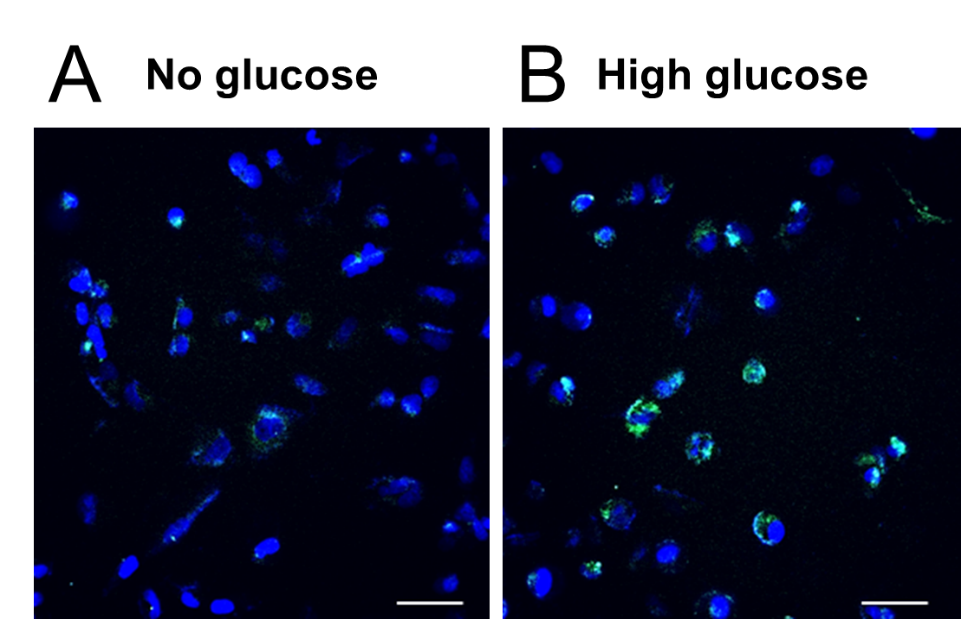


**Figure S7.** Validation of aptamer-modulated split GFP response to intracellular ATP using cell culture-induced ATP changes. ssAPC and ipGFP1-9 were transfected into (A) A549 cells with low ATP levels and (B) A549 cells with high ATP levels using Lipofectamine. The scale bar indicates 50 µm. The figure shows representative data of 3 independent experiments.

**
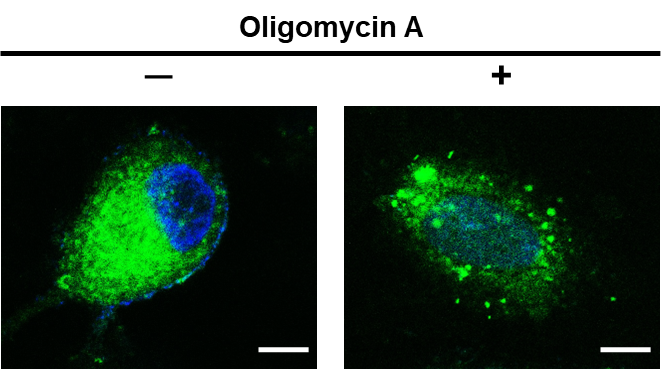
**
**Figure S8.** ATP depletion assay using Oligomycin A. An ATP depletion assay was performed using Oligomycin A to assess the reliability of ssAPC/ipGFP1-9 as an indicator of intracellular ATP levels under conditions of reduced ATP synthesis. Oligomycin A, an inhibitor of ATP synthase, was employed to induce ATP depletion in treated cells. Subsequent to treatment with ssAPC/ipGFP1-9 and Oligomycin A, a conspicuous decrease in the intensity of green fluorescence was noted compared to untreated controls. The scale bar indicates 10 µm. The figure shows representative data of 3 independent experiments.
